# TIGIT Induces (CD3+) T Cell Dysfunction in Colorectal Cancer by Inhibiting Glucose Metabolism

**DOI:** 10.3389/fimmu.2021.688961

**Published:** 2021-09-29

**Authors:** Qi Shao, Lei Wang, Maoling Yuan, Xiaohong Jin, Zhiming Chen, Changping Wu

**Affiliations:** ^1^ Department of Tumor Biological Treatment, The Third Affiliated Hospital of Soochow University, Changzhou, China; ^2^ Department of Chemotherapy, Affiliated Hospital of Nantong University, Nantong, China; ^3^ Department of Gastrointestinal Surgery, The Third Affiliated Hospital of Soochow University, Changzhou, China; ^4^ Department of Radiotherapy, Affiliated Hospital of Nantong University, Nantong, China; ^5^ Department of Oncology, The Third Affiliated Hospital of Soochow University, Changzhou, China

**Keywords:** TIGIT, colorectal cancer, immunotherapy, tumor microenvironment, CD3+ T cell

## Abstract

T-cell immunoglobulin and immunoreceptor tyrosine-based inhibitory motif domain (TIGIT) is an immunosuppressive receptor expressed on the surface of immune cells, suppressing immune responses by activating the intracellular negative regulatory signals. TIGIT plays an important role in the pathogenesis of various tumors, but its immune escape in colorectal cancer remains unclear. We found that the proportion of CD3^+^TIGIT^+^ T cells was increased in peripheral blood and cancer tissue in colorectal cancer patients when compared with the healthy donors. These cells exhibited functional defects, low proliferative activity, impaired cytokine production and reduced glucose metabolism. A strong association was also observed between the elevated TIGIT expression and poor prognosis in this cohort. In the *in vitro* co-culture assays of T cells and tumor cells, the suppressed glucose metabolic activity of T cells was reversed by TIGIT blockade. In addition, this blockade induced the apoptosis and reduced G2/M transit in tumor cells. The antitumor efficacy of TIGIT Ab therapy was further demonstrated in a human colorectal xenograft mice model while co-blockers of TIGIT and PD-1 exhibited synergistic suppressing effects on tumor growth. These results suggest that while TIGIT induces CD3^+^ T cell dysfunction in colorectal cancer, co-targeting TIGIT and PD-1 can lead to an effective antitumor response and may serve as a novel therapeutic strategy for colorectal patients.

## Introduction

Colorectal cancer has the third highest incidence and the second highest mortality rate according to the 2020 Global Cancer Statistics (1). Traditional treatment such as surgical resection, chemo-radiotherapy and targeted therapy has encountered development bottleneck, while tumor immunotherapy has emerged as a more promising therapeutic strategy.

As a core component of the immune response, T cells are present in the tumor microenvironment and determine the efficacy of anti-tumor immunotherapy (2). Immune checkpoints are signaling molecules on the surface of T cells that regulate their activity and participate in the immune response (3–5). It has been proved that tumor cells can escape from the immune surveillance by activating immune checkpoints which then release inhibitory signals (6–8). Immune homeostasis can be restored by introducing immune checkpoint-blocking antibodies, either alone or in combination therapy, which helps to reinvigorate T cell activation and proliferation (9–12). There is a growing awareness of the critical role of immune checkpoints in tumor immunotherapy. Although many new checkpoints on T cells’ surface have been reported in recent years, few of them have exerted promising result when used in colorectal cancer patients. Therefore, identifying a new immunotherapeutic and target is of great importance.

TIGIT (T-cell immunoglobulin and immunoreceptor tyrosine-based inhibitory motif domain), as a novel member of the CD28 family, is located on human chromosome 3. It contains an immunoglobulin variable region (IgV), a transmembrane region, and a tyrosine inhibitory motif region of the immune receptor (13–15). It is widely expressed on immune cells such as NK cells, activated T cells, memory T cells, Tregs, and even some cancer cells (13). TIGIT is a conservative molecule, and its homologous molecules have been found in many mammals (16). TIGIT, as a co-inhibitory receptor, binds to Polio Virus receptor (PVR, CD155) with the highest binding affinity, followed by PVRL2 (CD112) and PVRL3 (CD113) (13). It binds to the same site on PVRs with the affinity receptors CD226 which transmits positive co-stimulatory signals, or CD96 which transmits inhibitory signals (13, 17).

At present, the research of TIGIT has been conducted in a wide range of diseases, including infection, autoimmune diseases and tumors, with the latter one becoming a hotspot of research focus (17–20). It has been confirmed that TIGIT expression is upregulated in various tumors, and many studies have proven that the use of TIGIT antibody alone or synergized with others can achieve promising preclinical results (20, 21). Zhou (16)suggested that endogenous TIGIT mediated NK cells and CD8^+^ T cells malfunction were involved in the tumor promotion process. Furthermore, blocking TIGIT/PVR interaction with antibodies could induce anti-tumor effects by promoting tumor infiltration and restoring CD8^+^ T cell function. Josefsson (22)found that TIGIT and PD-1 were common co-inhibitory receptors expressed on CD8^+^ and CD4^+^ T effector memory cells in NHL patients. TIGIT and PD-1 together can be used to mark dysfunctional T cells, and co-inhibition of TIGIT and PD-1 could trigger robust anti-tumor responses in patients with NHL. These studies have undoubtedly revealed the role of TIGIT as a key checkpoint for tumors; however, little is known about whether TIGIT regulates the micro-environment in colorectal cancer patients and its specific mechanisms.

In this study, we found that the expression of TIGIT was upregulated on the T cell surface in colorectal cancer patients and had exhibited a significant relationship with clinical prognosis. Elevated TIGIT on CD3^+^ T cells led to functional defect and impaired glucose metabolism. We have also shown that antibody blockade of TIGIT could restore CD3^+^ T cell activity and inhibit tumor growth, which might suggest a promising target for colorectal cancer.

## Materials and Methods

### Clinical Participants

From September to December 2019, peripheral blood samples and tumor tissues were collected from 24 pathologically verified patients with colorectal cancer from the Third Affiliated Hospital of Soochow University (cohort 1). Peripheral blood was collected 1-3 days before the surgery. The exclusion criteria were (1) patients with infection and autoimmune diseases, (2) patients who had received chemotherapy or radiotherapy before surgery. Peripheral blood samples from 20 healthy donors (HDs) were used as controls. Informed consent was obtained from every patient. This study was approved by the Medical Ethics Committee of the Third Affiliated Hospital of Soochow University and was conducted in accordance with ethical guidelines: Declaration of Helsinki.

### Immunofluorescence

Tissue microarrays (HRec-Ade180Sur-03, Shanghai Outdo Biotech Co.) were constructed using formalin-fixed, paraffin-embedded carcinoma tissues matched to adjacent normal tissues collected from 90 colorectal cancer cases (cohort 2). Immunofluorescence was performed following an established protocol. Briefly, tissue microarrays were dewaxed, rehydrated through graded ethanol, and incubated with 3% hydrogen peroxide for 30 mins. Antigen retrieval was performed by heating the sections to 95 °C in 0.01 M citrate buffer (pH6.0) for 15 mins. Slides were then washed in PBS for 15 mins, treated with 10% normal horse serum for 30 mins and incubated with the primary antibodies, including Alexa Fluor 549 mouse anti-CD3 (BioLegend, San Diego, USA) and DyLight 488rabbit anti-TIGIT (BioLegend, San Diego, USA). After staining, the slides were mounted with ProLong Gold Antifade Mountant with 4’,6-diamidino-2-phenylindole (DAPI, Thermo Fisher, USA). The images were taken using the Leica Aperio System (Leica, USA).

### Cell Isolation

Peripheral blood mononuclear cells (PBMCs) were isolated with Ficoll–Hypaque by density gradient centrifugation within 2 hours of peripheral blood sample collection. Fresh colorectal tumor tissues were minced and digested within 2h of surgery. Digested cells were filtered through a 70 um nylon mesh and washed with PBS. Native CD3^+^ T cells were purified from PBMC by negative selection using the EasySep human total or native CD3^+^ T Cell EnrichmentKits (STEMCELL Technologies Inc. USA). CD3^+^TIGIT^+^T cells and CD3^+^TIGIT^-^T cells were sorted using a BD FACS Influx (BD Biosciences, USA).

### Flow Cytometry

PBMCs and tumor tissues isolated from colorectal cancer patients or HDs were stained with the following antibodies: PC5 conjugated anti-CD3, Ac7 conjugated anti-CD4, PE-Cy7 conjugated anti-CD8, APC conjugated anti-TIGIT, PE conjugated anti-Tim-3, FITC conjugated anti-PD-1, PE-Cy7 conjugated anti-INF-ϒ, V670 conjugated anti-IL-2, PC7 conjugated anti-Granzyme B, and V450 conjugated anti-IL10 antibodies. Samples were analyzed using BD FACS ARIA (BD Biosciences).

### Glucose Metabolism

Glucose consumption reflects the metabolic activity of many cells. 2-deoxyglucose (2-DG) is a glucose analog and widely used to assess glucose uptake. Similar to glucose, 2-DG can be taken up by glucose transporters and then metabolized to 2-DG-6-phosphate (2-DG6P). 2-DG6P is oxidized, resulting in the generation of NADPH which can be determined by an enzymatic recycling amplification reaction. T cells (2x10^5^/well, 50ul) were stimulated with aCD3/CD28 (5 ug/mL) for 8 hours. The cells were washed 3 times with PBS and then glucose starved with 100 mL Krebs-Ringer-phosphate-HEPES buffer containing 2% BSA for 40 min. The cells were then stimulated with or without 1 mmol/L insulin for 20 minutes. 10 uL10umol/L2-DG was added into the cells for 20 mins. Glucose metabolism was analyzed according to the manufacturer’s instructions of the 2-DG Assay Kit (Sigma-Aldrich, USA).

### Lactate and Pyruvate Production Assay

CD3^+^TIGIT^+^ T or CD3^+^TIGIT^-^ T cells (2*10^5^/well) were stimulated with aCD3/CD28 for 8 hours and then cultured with fresh complete medium containing glucose. Lactate concentrations and pyruvate concentrations were analyzed according to the manufacturer’s instructions of the Lactate Assay Kit (Abnova, USA) and Pyruvate Assay Kit (Abnova, USA).

### Extracellular Acidification Rate

Measurement of extracellular acidification rate (ECAR) was performed in a 96-well XF Extracellular Flux Analyser (Seahorse Biosciences). Sensor cartridge injection compounds or XF Base Media controls were injected in a total volume of 25 μl. Injection compound concentrations were as follows: glucose (5 mM), oligomycin (OLIGO) (mitochondrial ATP synthase inhibitor) (2.5 μM), 2-DG (100 mM). Sensor cartridges (96-well) were hydrated for a minimum period of 12 h with XF Calibrant solution according to the manufacturer’s instructions (Seahorse Biosciences, North Billerica, MA, USA). XF Assay Base Media (Seahorse Biosciences) was supplemented with l-glutamine (2 mM) for ECAR measurement. Glycolysis parameters [glycolysis, glycolytic capacity (GC), glycolytic reserve (GR)] were calculated as follows: Glycolysis: (Maximal rate measurement after glucose injection through measurement prior to oligomycin (OLIGO) injection) minus (measurement prior to glucose injection); GC: (Maximal rate measurement after OLIGO injection through measurement prior to 2-DG injection) minus (measurement prior to glucose injection); GR: GC minus glycolysis (23).

### T-Cell Proliferation Assays

In T-cell proliferation assays, cells (5*10^5^/well) were seeded in 96-well plates, labeled with 5mmol/L carboxy fluorescein diacetate succinimidyl ester (CFSE), and stimulated with aCD3/CD28(5 ug/mL) at 37°C with 5% CO_2_ for 4 days. Cells were collected, and the dilution of intracellular CFSE caused by proliferation was calculated using BD FACS ARIA (BD Biosciences).

### Cell Culture

Human HCT-116 colorectal cancer cells were cultured in RPMI-1640 medium (GIBCO, Grand Island, USA) supplemented with 10% FBS (fetal bovine serum) and streptomycin/penicillin at 37°C with 5% CO_2_ under fully humidified conditions, while murine MC38 colorectal cancer cells were cultured in DMEM medium (GIBCO, Grand Island, USA) under the same conditions. CD3^+^ T cells stimulated with aCD3/CD28 were sorted and co-cultured with HCT-116 cells at a ratio of 5:1 in 24-well plates for 2 days. Then 5 mg/mL anti-TIGIT antibodies (BPS Biosciences, USA) or isotype controls were added to the co-culture system. T cells were collected to measure, their activity and the key markers in the metabolic pathways by flow cytometry, RT-PCR and western blot. HCT-116 cells were collected to determine the apoptosis and cell cycle distribution.

### Real-Time PCR

Total RNA was isolated using Trizol reagents (Thermo Fisher, Inc., USA) according to the manufacturer’s protocol. cDNA was obtained using a 1μg total RNA reverse transcription kit (Life Technologies, USA). The qPCR reaction was performed using the SYBR Green PCR Master Mix kit (Takara, Japan) through the ABI 7900 PCR system (Applied Biosystems, USA). The data were quantified according to the ^2-ΔΔ^CT method. The mRNA expression levels of cultured cells were normalized to GAPDH, respectively. Primer sequences are as follows:Glut1 (Glucose transporter 1),5’-ACTGCTGGAGCAGCTACCCT-3’ and 5’-GAAGCCTGCAACGGCAATGG-3’;HK1(hexokinase 1), 5’-CTGTTACGTCGGCGCTGCTA-3’ and 5’-AGAGCGCCATTGTCATCGGG-3’;HK2(hexokinase 2), 5’-CTGTTACGTCGGCGCTGCTA-3’ and 5’-AGAGCGCCATTGTCATCGGG-3’; PFK(phosphofructokinase), 5’-TCCGATTTCAGCATCGGGGC-3’ and 5’-CAGGTAGCCACAGTAGCCGC-3’; GAPDH,5’-GCGGGGCTCTCCAGAACAT-3’ and 5’-TCCACCACTGACACGTTGGC-3’.

### Western Blot

The total protein was extracted using a mixture of RIPA lysis buffer and protease inhibitor. Proteins were quantitated using a BCA protein assay kit (Pierce, Keygen Biotech, China), then isolated with SDS-PAGE gel and transferred to PVDF membrane (Millipore, Darmstadt, Germany). The membrane was sealed with Tris buffer containing 0.1% Tween-20 and 5% skimmed milk at 4°CC. Rabbit anti-p-Akt (Ser473), Akt (1:1000; CST, Denver, MA, USA), mTOR (S2448) and p-mTOR (1:1000; CST, DANVERS, MA) were incubated with membrane overnight and then treated with HRB-conjugated secondary antibodies (1:10000; CST, Denver, MA, USA). The signal is detected by the Enhanced Chemiluminescence Detection System (Tennon5200, Shanghai, China), following the manufacturer’s instructions.

### Xenotransplant Models

MC38 cells (5×10^6^ cells/mouse) were injected subcutaneously into the right flank of B6J mice (n=6, male; 4-week). When the tumor volume reached 100 mm^3^, 24 tumor-bearing mice were evenly divided into 4 groups. The TIGIT antibody (10917-MM52, Sinobiology, Beijing) and PD-1 antibody (10377-M94, Sinobiology, Beijing) were both administrated in a dose of 10 mg/kg *via* tail-vein injection three times a week. The tumor volume of mice was measured every 5 days and mice were sacrificed after 30 days with the xenograft tumors harvested for further analysis. All experimental procedures were performed following the NIH Guide for Care and Use of Animals and were approved by the Institutional Animal Care and Use Committee.

### Apoptosis Assay and Cell-Cycle Analysis

Cells (1*10^6^) were collected and incubated with the solutions of Annexin V and propidium iodide (PI) according to the apoptosis detection kit (BD Biosciences Pharmingen). For cell-cycle analysis, cells were fixed in 70% ice-cold ethanol at 4°C overnight and then stained in a mixed solution (200 mg/mL PI, 0.1% sodium azide, 0.1% Triton X-100, and 10 mg/mL RNase). The apoptotic cells and cell-cycle distribution were detected by a FACSCalibur flow cytometer.

### Statistical Analysis

Three independent experiments were conducted for each treatment, and all data were expressed as mean ± standard deviation. The student’s t-test was only used for difference analysis between two groups, and one-way analysis of variance (ANOVA) was used when three or more groups were compared. *P*<0.05 was considered statistically significant. SPSS version 22.0 (SPSS, Chicago, IL, USA) software was used for statistical analysis of clinical samples.

## Results

### Upregulated TIGIT Expression on CD3^+^ T Cells Correlated With Poor Survival in Patients With Colorectal Carcinoma

**Figure 1:**
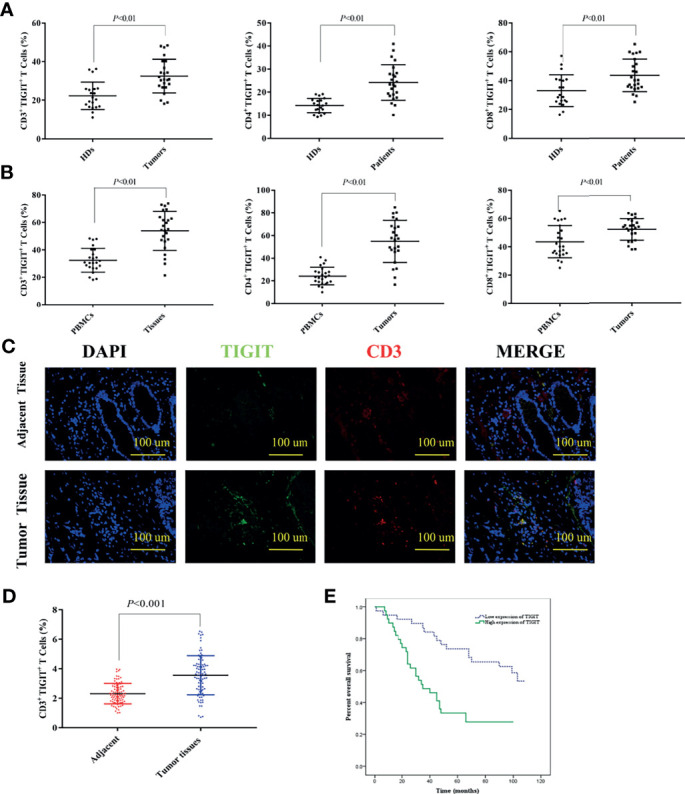
Upregulated TIGIT expression in patients with colorectal cancer predicts poor prognosis. **(A)** Percentage of CD3^+^TIGIT^+^ T, CD4^+^TIGIT^+^ T and CD8^+^TIGIT^+^ T cells in PBMCs of HDs and colorectal cancer patients. **(B)** Percentage of CD3^+^TIGIT^+^T, CD4^+^TIGIT^+^ T and CD8^+^TIGIT^+^ T cells in PBMCs and tumor tissues of colorectal cancer patients. **(C)** Double fluorescence staining of colorectal cancer detecting CD3^+^TIGIT^+^T cells. Immunofluorescent staining of TIGIT^+^ cells (in green) and CD3^+^ cells (in red). The merged picture indicated double-positive cells located in the stroma. The image was shown in original magnification of 200×. **(D)** The quantitative analysis of CD3^+^TIGIT^+^ T cell in tissue microarrays. **(E)** Kaplan-Meier curve for an independent set of 77 colorectal patients (*P* < 0.01).

TIGIT expression in T cells from colorectal cancer patients was evaluated by flow cytometry in cohort 1. The results suggested that the percentage of CD3^+^TIGIT^+^ T cells(32.50 ± 8.74) in peripheral blood of colorectal cancer patients was significantly increased compared with that of HDs (22.28 ± 7.16)(*P*<0.01). There were also significant differences between the percentage of CD4^+^TIGIT^+^ T cells (24.21 ± 7.72) *vs.* HDs (14.20 ± 3.13) and the percentage of CD8^+^TIGIT^+^ T cells(43.57 ± 11.32) *vs.* HDs (33.09 ± 11.03) in peripheral blood (P<0.01). It was noticed that the percentage of CD3^+^TIGIT^+^ T(53.89 ± 14.05), CD4^+^TIGIT^+^ T(54.88 ± 18.56) and CD8^+^TIGIT^+^ T cells (52.03 ± 7.65) were significantly increased in tumor tissues when compared with peripheral blood samples (**Figures 1A, B**). The infiltration of CD3^+^TIGIT^+^ T cells was further examined in cohort 2 of 90 matched sets of colorectal carcinoma tissues and adjacent normal tissues using tumor tissue microarray analysis. As shown in **Figure 1C**, double fluorescence staining revealed a higher degree of CD3^+^TIGIT^+^ T cell infiltration in colorectal carcinoma tissues than adjacent normal tissues, which was supported by the quantitative analysis (**Figure 1D**). To assess the prognosis value of TIGIT, the relationship between TIGIT expression and clinic pathological parameters or survival was investigated in 77 evaluable cases. TIGIT expression was notably associated with the pathological stage (*P*<0.001, **Table 1**). In addition, both univariate and multivariate survival analyses suggested that TIGIT was an independent adverse prognostic factor for colorectal cancers (*P*<0.05, **Table 1**). Simultaneously, we found that a higher CD3^+^TIGIT^+^ cells expression level was associated with a poorer prognosis (*P*< 0.01, **Figure 1E**).

**Table 1:** Univariate and multivariate statistics of the prognostic value of clinic pathological parameters and TIGIT expression level for survival in colorectal cancer.

Parameters	low	high	χ^2^	*P*	Univariate		Multivariate		
	N = 38	N = 39			*P*	95% CI	*P*	95% CI
**Gender**			0.314	0.575	0.735	0.605-2.040	0.862	0.538-2.100	
Male	23	26							
Female	15	13							
**Age(years)**			0.106	0.744	0.033^*^	1.057-3.883	0.197	0.787-3.185	
≤60	16	15							
>60	22	24							
**Size(cm)**			0.312	0.576	0.876	0.575-1.914	0.764	0.577-2.117	
≤5	21	24							
>5	17	15							
**T stage**			2.503	0.114	0.489	0.546-3.543	0.449	0.546-3.921	
T1+T2	3	8							
T3+T4	35	31							
**Lymph node metastasis**			1.597	0.206	0.009^*^	1.220-4.079	0.036^*^	1.046-3.911	
Negative	23	18							
Positive	15	21							
**TNM stage**			1.597	0.206	0.009^*^	1.220-4.079	0.036^*^	1.046-3.911	
I+II	23	18							
III+IV	15	21							
**Pathological stage**			32.195	0.000^*^	0.000^*^	2.510-9.836	0.027^*^	1.110-5.820	
I+II	37	26							
III	1	13							
**TIGIT expression**	38	39			0.001^*^	1.545-5.522	0.030^*^	1.081-4.803	

Results of univariate and multivariate analyses using the log-rank test and the Cox proportional hazards model. In the univariate analyses, age, lymph node metastasis, TNM stage, pathological stage, and TIGIT expression were significantly associated with poor survival (P<0.05). In the multivariate analyses, lymph node metastasis, TNM stage, pathological stage, and TIGIT expression (P<0.05). CI, confidence interval. *P < 0.05, Statistically significant.

### TIGIT Overexpressed CD3^+^T Cells Exhibited a Defect in Proliferation and Cytokines Production in Human Colorectal Cancer Patients

**Figure 2:**
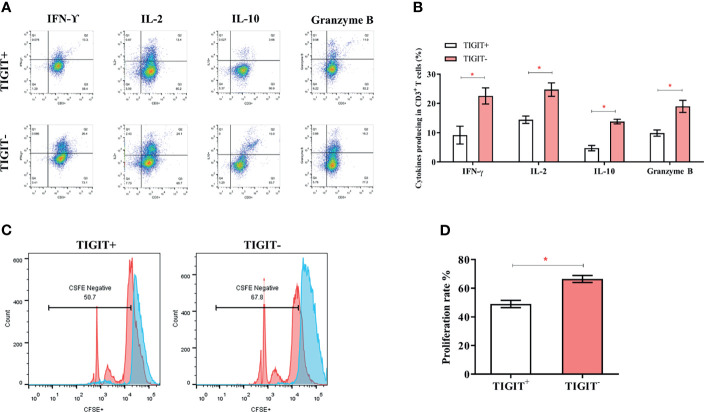
TIGIT impairs CD3^+^T cells’ function and leads to a reduced proliferation rate. The frequency of IFN-γ, IL-2, IL-10, and GranzymeB secreting of CD3^+^T cells was detected by FACS. **(A)** Typical flow cytometry images in CD3^+^TIGIT^+^ T cells and CD3^+^TIGIT^-^ T cells. **(B)** Data was shown from three experiments conducted in 6 samples. **(C)** Cells were stained with CFSE, and proliferation rates were detected by FACS. Representative histograms are shown. **(D)** Proliferation of CD3^+^TIGIT^+^ T cells and CD3^+^TIGIT^-^ T cells. (*P < 0.05).

TIGIT expression is correlated with T cell dysfunction (16, 19, 22). We then investigated whether these TIGIT overexpressed CD3^+^T cells had altered cell function in colorectal cancer patients. CD3^+^TIGIT^+^ or CD3^+^TIGIT^-^ T cells were sorted from patients’ PBMCs by flow cytometry. The secretion of INF-γ, IL-2, IL-10 and Granzyme B was significantly suppressed in CD3^+^TIGIT^+^T cells than in CD3^+^TIGIT^-^ T cells(**Figures 2A, B**). In addition, the proliferation rate of CD3^+^TIGIT^+^T cells was also reduced (**Figures 2C, D**). We also analyzed the relation between clinic pathological parameters and the percentage of CD3^+^TIGIT^+^PD-1^+^ T cells or CD3^+^TIGIT^+^Tim-3^+^ T cells in cohort 1 (**Table 2**). The patients were divided into high and low groups according to the median proportion of CD3^+^TIGIT^+^, CD3^+^TIGIT^+^PD-1^+^ or CD3^+^TIGIT^+^Tim-3^+^ T cells. The results revealed that the percentage of CD3^+^TIGIT^+^PD-1^+^ T cells was significantly associated with the node stage (*P*<0.05). However, such a correlation was not found for CD3^+^TIGIT^+^Tim-3^+^ T cells.

**Table 2:** Correlations between immune markers expressions and clinic pathologic information.

Characteristics	CD3^+^TIGIT^+^Mean 53.90 Range21.40-73.80		*P*	CD3^+^TIGIT^+^PD-1^+^ Mean 21.33 Range7.21-46.00		*P*	CD3^+^TIGIT^+^Tim-3^+^ Mean 17.88 Range 5.94-34.50		*P*
	low	high		low	high		low	high	
**Gender**			0.643			0.643			0.395
Male	7	10		10	7		7	10	
Female	3	4		4	3		4	3	
**Age(years)**			0.582			0.418			0.375
≤60	4	5		6	3		6	4	
>60	6	9		8	7		5	9	
**Size(cm)**			0.473			0.185			0.353
≤ 5	4	7		8	3		6	5	
> 5	6	7		6	7		5	8	
**T stage**			0.527			0.473			0.647
1-3	5	8		7	6		6	7	
4	5	6		7	4		5	6	
**N stage**			0.643			0.010^*^			0.605
1-3	7	10		7	10		8	9	
≥4	3	4		7	0		3	4	
**Vascular invasion**			0.494			0.171			0.122
yes	7	11		9	9		6	11	
no	3	3		5	1		5	2	
**MSI**			0.670			0.670			0.283
MSI-H	1	1		1	1		0	2	
MSI-I	9	13		13	9		11	11	

*P < 0.05, Statistically significant.

### TIGIT Overexpressed T Cells Had an Impairment in Glucose Uptake and Metabolism in Colorectal Cancer Patients

**Figure 3:**
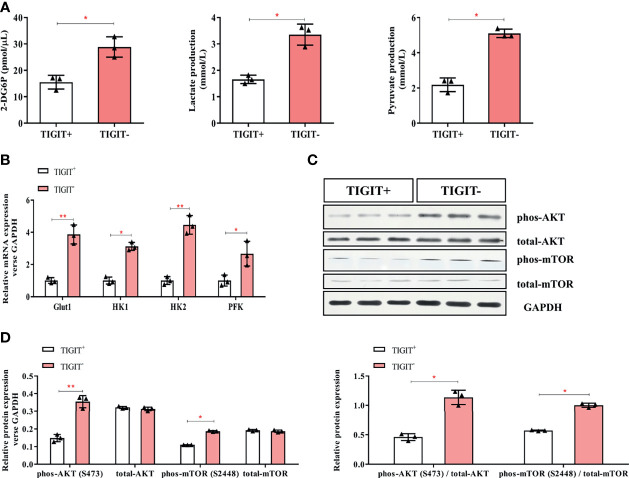
Glycometabolism of TIGIT^+^CD3^+^ T cells is decreased in colorectal cancer patients. TIGIT^+^CD3^+^ T cells were sorted from PBMC of colorectal cancer patients. **(A)** The concentration of 2-DG6P, Lactate and Pyruvate was reduced in TIGIT^+^CD3^+^ T cells compared to TIGIT^-^CD3^+^ T cells. **(B)** Glut1, HK1, HK2, and PFK were measured by RT-PCR and mRNA expression was quantified. Glut1, HK1, HK2, and PFK elevated in TIGIT^+^CD3^+^ T cells. **(C)** Representative blots of western blotting were shown. **(D)** Western blotting analysis was performed to determine the phosphorylation levels of the AKT/mTOR pathway. The results were normalized to GAPDH expression and presented as relative protein expression. *P < 0.05; **P < 0.01.

Glucose uptake and metabolism of T cells play a critical role in maintaining its function activated. Oxidative phosphorylation and glycolysis are crucial for T cells to obtain energy (23).To determine the metabolic activity changes of T cells, we first measured the content of 2-DG6P and found that it was significantly reduced in CD3^+^TIGIT^+^T cells (15.53 pmol/ul) compared with CD3^+^TIGIT^-^ T cells (28.83 pmol/ul, *P*<0.05, **Figure 3A**). Then we measured the concentrations of lactate and pyruvate. We found that the lactate concentrations was significantly decreased in CD3^+^TIGIT^+^ T cells (1.66 mmol/ul) compared with CD3^+^TIGIT^-^ T cells (3.52 mmol/ul, *P*<0.05, **Figure 3A**). In addition, the pyruvate concentrations was significantly lower in CD3^+^TIGIT^+^ T cells (2.18 mmol/ul) than CD3^+^TIGIT^-^ T cells (5.10 mmol/ul, *P*<0.05, **Figure 3A**).

Glut1 and other key enzymes for initiating the glucose metabolic process were then detected by qPCR. The results showed that Glut1, HK1, HK2, and PFK were significantly lower in CD3^+^TIGIT^+^ T cells (**Figure 3B**). In addition, the AKT/mTOR pathway regulates glycolysis and phosphorylation of AKT and mTOR promotes cell proliferation and activation (20). We found that the phosphorylation level of the Akt/mTOR pathway in CD3^+^TIGIT^+^T cells was significantly lower than those in CD3^+^TIGIT^-^T cells by western blot (**Figures 3C, D**). All the differences were statistically significant (*P*<0.05). Taken together, these findings demonstrated that TIGIT overexpressed T cells had impaired glucose uptake and metabolism in colorectal cancer patients.

**Figure 4:**
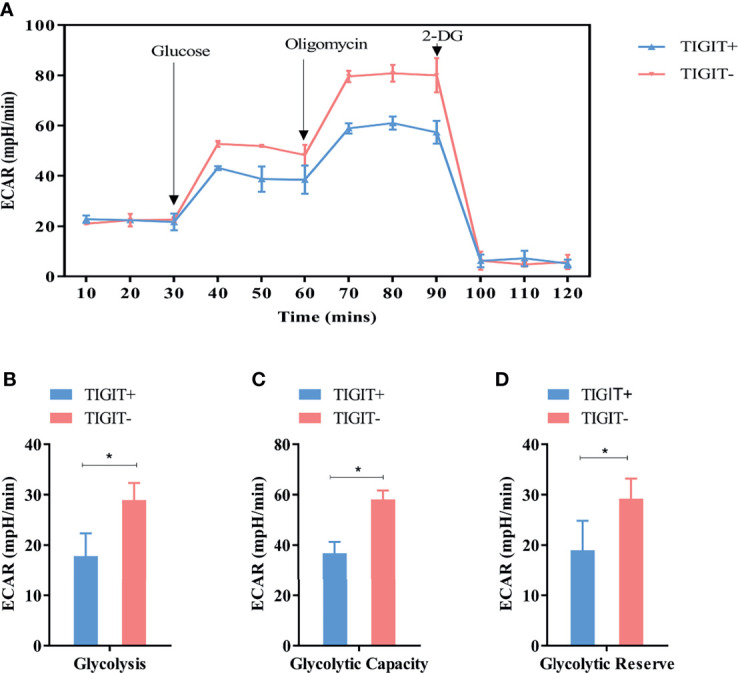
Measurement of ECAR in TIGIT^+^ T and TIGIT^-^ T cells. **(A)** Kinetic ECAR response of TIGIT^+^ T and TIGIT^-^ T cells. **(B–D)** Measurement of Glycolysis, Glycolytic Capacity and Glycolytic Reserve. *P < 0.05.

Extracellular acidification rate assay was designed to assess the glycolysis function of cells. Glucose was added to determine the glycolysis level under glucose saturation, and oligomycin was added to inhibit mitochondrial respiration to determine the GC. These parameters were used to calculate the GR. The results suggested that CD3^+^TIGIT^+^ T and CD3^+^TIGIT^-^T cells had different ECAR responses in terms of glycolysis rates, GR and GC. The glycolysis, GR and GC were significantly reduced in CD3^+^ T cells (*P*<0.001, **Figure 4**).

### TIGIT Blockade Reversed Impaired TIGIT^+^ T Cell Metabolism and Induced Apoptosis of Colorectal Cancer Cells

**Figure 5:**
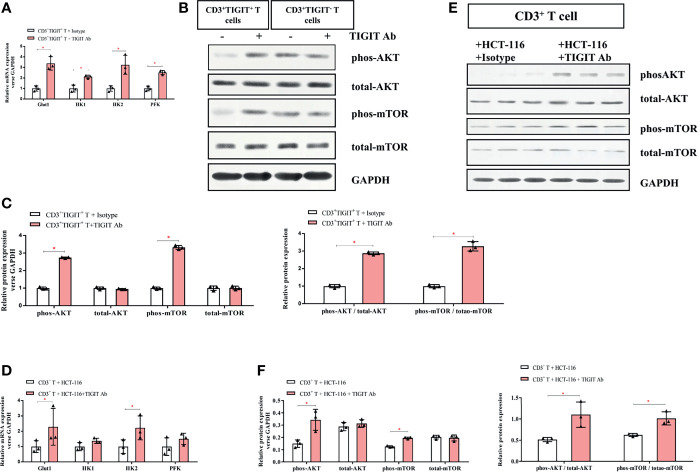
TIGIT blockade restores the glucose metabolic activity of T cells. **(A)** mRNA expression of Glut1, HK1, HK2, and PFK elevated in TIGIT Ab group compared with the control group in CD3^+^TIGIT^+^ T cells. **(B)** Representative western blots for the control group and TIGIT Ab group in CD3^+^TIGIT^+^ T cells and in CD3^+^TIGIT^-^ T cells. **(C)** Western blotting analysis of the phosphorylation levels of the AKT/mTOR pathway in the control group and TIGIT Ab group in CD3^+^TIGIT^+^ T cells. Then, CD3^+^ T cells stimulated with aCD3/CD28 were co-cultured with HCT-116 at a ratio of 5:1. In the co-culture system, isotype control and TIGIT Ab were respectively added as the control group and experimental group. **(D)** mRNA expression of Glut1 and HK2 elevated in the experimental group**. (E)** Representative blots of the control group and the experimental group were shown. **(F)** Western blotting analysis of the phosphorylation levels of the AKT/mTOR pathway in the control group and the experimental group. *P < 0.05.

To further explore the role of TIGIT in T cell metabolism, a TIGIT antibody was used to block TIGIT. CD3^+^TIGIT^+^T and CD3^+^TIGIT^-^T cells were sorted from colorectal cancer patients’ PBMCs. Isotype and TIGIT antibody were added to CD3^+^TIGIT^+^T cells or CD3^+^TIGIT^-^T respectively as control group and TIGIT Ab group. As shown in **Figure 5A**, expression of glucose metabolism-associated enzymes (Glut1, HK1, HK2, and PFK) was significantly increased in CD3^+^TIGIT^+^ T cells treated with TIGIT antibody. Moreover, phosphorylation levels of Akt and mTOR were both enhanced in CD3^+^TIGIT^+^ T cells treated with TIGIT antibody (**Figures 5B, C**). However, in the CD3^+^TIGIT^-^T cells, we didn’t find a significant increase in the expression of Glut1, HK1, HK2 and PFK, and enhanced Akt and mTOR phosphorylation levels (**Figure S1** and **Figure 5B**). Then we isolated CD3^+^ T cells from PBMCs of colorectal cancer patients and co-cultured them with colorectal cancer cell lines of HCT-116. Isotype and TIGIT antibody were added to the co-culture system as the control group and experiment group. After adding the TIGIT antibody into the co-culture system, the expression levels of Glut1 and HK2 were upregulated than the control group. However, there were no changes in HK1 and PFK(**Figure 5D**). Simultaneously, increased AKT and mTOR phosphorylation expression in CD3^+^ T cells was confirmed at the protein level by Western blotting (**Figures 5E, F**).

**Figure 6:**
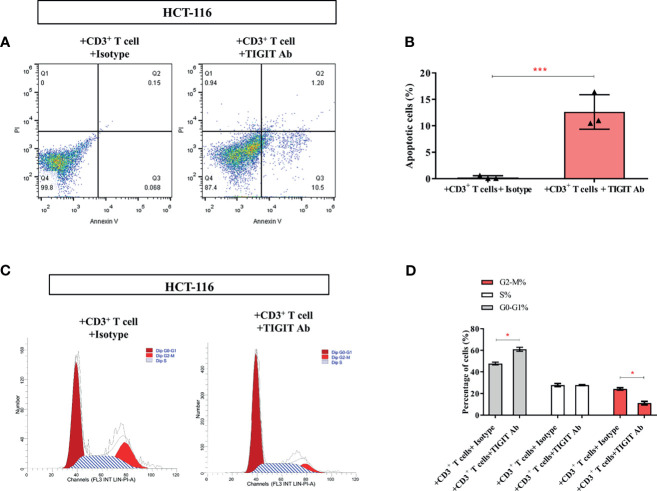
TIGIT inhibits the proliferation of colorectal cancer cells. In the co-culture system, **(A, B)** Apoptosis of HCT-116 was detected cell apoptosis by Annexin V assay. **(C, D)** Flow cytometry was applied to determine cell-cycle distribution. *P < 0.05; ***P < 0.001.

As the above data showed that TIGIT blockade reversed impaired T cell metabolism, we then investigate if TIGIT blockade could affect colorectal cancer cells. As shown in **Figures 6A, B**, the percentage of apoptotic HCT-116 cells was dramatically increased in the presence of TIGIT antibody (0.25% *vs.* 12.63%, *P*<0.05). Furthermore, the percentage of G2-M cells was dramatically decreased by TIGIT blockade (24.31% *vs.* 11.14%, *P*<0.05, **Figures 6C, D**). These results demonstrated that TIGIT blockade inhibited cell proliferation and induced apoptosis in HCT-116 colorectal cancer cells cocultured with CD3^+^ T cells.

Taken together, these findings indicated that the use of TIGIT antibody could restore the metabolic activity of TIGIT^+^ T cells and suppress tumor growth in the coculture assay.

### Combined Inhibition of TIGIT and PD-1 Signals Synergistically Suppressed Tumor Progression *In Vivo*

**Figure 7:**
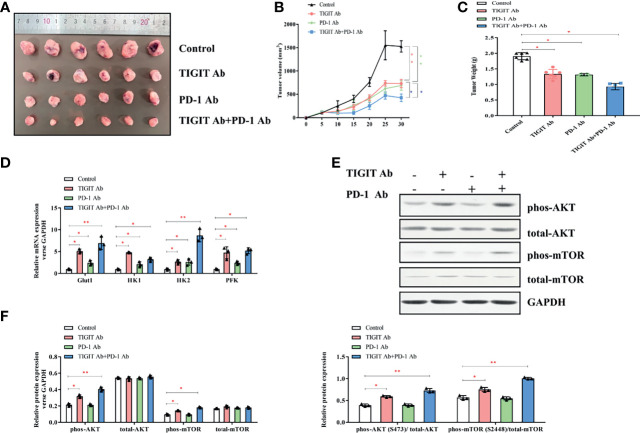
Impact of TIGIT and/or PD-1 antibody on tumor growth *in vivo*. **(A)** Tumors were removed and collected from B6J mice injected with TIGIT and/or PD-1 antibody. **(B)** The tumor volume was analyzed every 5 days. **(C)** The tumor weight was measured 30 days after tumor transplantation. **(D)** RT-PCR was applied to quantify the mRNA levels of Glut1, HK1, HK2, PFK, and GAPDH. **(E)** Representative blots of each group. **(F)**, Western blotting analysis was performed to determine the expression of phos-AKT, total-AKT, phos-mTOR, total-mTOR. *P < 0.05; **P < 0.01.

Given the potential interaction between TIGIT and PD-1, we then assessed the antitumor effects of combined inhibition of TIGIT and PD-1 signals using a xenograft B6J mouse tumor model derived from MC38 colon adenocarcinoma cells. Mice were treated with TIGIT antibody, PD-1 antibody, or TIGIT combined with PD-1 antibodies. As shown in **Figures 7A–C**, while tumor burden (volumes and weight) were significantly reduced after anti-TIGIT or anti-PD-1 treatment, a further tumor inhibition was observed in mice treated with both anti-TIGIT and anti-PD-1. Furthermore, the expression of metabolism-associated genes (Glut1, HK1, HK2, and PFK) was further elevated in the mice that received both anti-TIGIT and anti-PD-1 treatments (**Figure 7D**). Western blot analysis revealed that phosphorylation of AKT and mTOR in tumor infiltrated TIGIT^+^ T cells was significantly activated by anti-TIGIT plus anti-PD-1 treatments (**Figures 7E, F**).

Taken together, these results indicated that the combination of TIGIT and PD-1 blockade had a synergistic effect in inhibiting tumor progression compared with TIGIT or PD-1 blockade alone.

## Discussion

To date, an increasing amount of studies have been conducted focusing on immune checkpoint targeting tumor microenvironment. One of the mechanisms of cancer immune escape is *via* the activation of immunosuppressive signaling pathways, among which, the PD-1/PD-L1 pathway and CTLA-4 pathway have been extensively studied (24). The successful application of anti-PD-1 and anti-CTLA-4 antibodies in tumor therapy provides a promising opportunity for tumor immunotherapy. With the deepening research on the immunosuppressive receptors on T cells, many studies have confirmed that the immunosuppressive receptor TIGIT expressed on the cell membranes of T cells and NK cells can bind to the ligand on the tumor-specific target cells to inhibit the function of the immune system (18, 20, 21). In the current study, we found that the percentage of CD3^+^TIGIT^+^T cells was significantly increased in the peripheral blood and tumor tissues of patients with colorectal carcinoma. In addition, such TIGIT overexpression was correlated with an unfavorable prognosis and tumor progression. While these TIGIT positive T cells showed immune dysfunction and reduced glycometabolic activity, the treatment with TIGIT antibody could reverse the immune failure of these cells, inhibit tumor progression and induce tumor cell apoptosis.

Many important immune molecules and their receptors are present on the surface of T cells, playing a critical role in the activation, differentiation, and proliferation of T cells. TIGIT is one of the immune checkpoint molecules. We analyzed the relationship between clinical features and TIGIT. In tissue microarrays, the clinic pathological analysis indicated that TIGIT was highly expressed in cancer tissues than adjacent tissues, and it was notably related to the pathological stage. Besides, it was suggested that TIGIT was an independent adverse prognostic factor in both univariate and multivariate analyses. Simultaneously, in blood samples, CD3^+^T cells co-expressed by TIGIT and PD-1 were associated with the lymph node stage (*P*<0.05). Additionally, the secretion of active cytokines and proliferation rate of TIGIT^+^T cells was dramatically reduced. However, in this study, TIGIT caused the increase of immunosuppressive factor IL-10. Almost all lymphocytes can synthesize IL-10, mainly by mononuclear macrophages and T cells. Nearly all mononuclear macrophages are the target cells of IL-10 inhibition. However, there are a wide variety of T cells, and the mechanism of IL-10 involvement is very complex. T cells stimulated and activated ERK1 and ERK2 MAP kinases to secrete IL-10. TIGIT can inhibit the production of IL-2 and INF-γ by T cells. IL-2 reduces IL-10 secretion through signal transduction and inhibition of the transcription factor STAT5. In addition, decreased INF-γ resulted in decreased ERK1 activity, which further inhibited IL-10 secretion. These may be the reasons why TIGIT causes the increase of immunosuppressive factor IL-10. Taken together, we infer that TIGIT may activate the inhibitory signaling pathway, impede the growth of immune cells and induce the T cells dysfunction. TIGIT negatively regulates the human immune function, promotes the proliferation and escape of tumor cells, and accelerates the progression of colorectal cancer.

In the tumor microenvironment, both tumor cells and immune cells can be metabolically reprogrammed to adapt to the microenvironment of low oxygen, acid, and low nutrition (25). Changes in the components of various cells and extracellular matrix in the microenvironment can promote the initiation and development of tumors, and lead to the differential levels of sugars, lipids, amino acids, and other related metabolites, namely metabolic reprogramming (26, 27). Understanding the microenvironment can provide new insights into the pathogenesis and early diagnosis of colorectal cancer, potentially leading to better treatment and prognosis strategies. Glucose is the most required nutrient for tumor cells. It is also a vital energy substance for the activation and function of T cells. Aerobic glycolysis metabolism in tumor-infiltrating T cells is not only necessary for cellular energy but also conducive to the synthesis of intermediates and signal transduction (28). Warburg effect is an abnormal feature of cancer cells and immune cells, that is, in the tumor microenvironment, some cells rely on glycolysis instead of oxidative phosphorylation to achieve energy metabolism under aerobic conditions (29). On the metabolite level, the Warburg effect mainly showed decreased glucose concentration and an increase in lactic acid production. Glut1 is the key to initiate the Warburg effect, and HK1/2 is the key enzyme in glycolysis. In this study, we found abnormal lower glucose metabolism in TIGIT^+^ T cells. The levels of Glut1 and several key enzymes in glucose metabolism were decreased in TIGIT^+^ T cells compared with TIGIT^-^ T cells. In addition, the phosphorylation of AKT/mTOR metabolic pathway was downregulated. Fortunately, the TIGIT antibody reversed this phenomenon. These results suggest that TIGIT can cause T cell energy utilization disorder in the tumor microenvironment of patients with colorectal cancer, and induce T cell dysfunction. TIGIT antibody can restore the T cells effectors function and metabolic activity, which may be a potential treatment for colorectal cancer.

In the past decade, immunotherapy has provided the possibility for the clinical treatment of cancers. However, most patients did not benefit from the immunotherapy. It may be that nutrients and metabolites in the tumor microenvironment can regulate the fate of peripheral immune cells (30). The high metabolism of cancer cells leads to the lack of nutrition and the accumulation of metabolites. At the same time, the energy utilization of immune function cells is impaired (31). In the tumor microenvironment, different metabolic patterns and nutrient-sensing mechanisms jointly regulate the response of immune cells to nutrients (32). Moreover, negative immune checkpoint molecules are often used by tumor cells to evade immune surveillance. The metabolic disorder of tumor cells will further affect the expression of cell surface markers, thus interfering with immune surveillance. To perform its necessary functions, immune cells must be metabolically adapted to the changes of the microenvironment. Further understanding of the metabolic reprogramming and the interaction between the metabolism of infiltrating T cells and tumor cells will provide a theoretical basis for the subsequent development of tumor therapy. In our study, we used the co-culture system of T cells and colorectal cancer cells to construct the tumor growth environment *in vitro*. TIGIT blockage restarted the metabolism pathway and increased the phosphorylation of the Akt/mTOR pathway in CD3^+^ T cells, resulting in restoring the metabolic function of T cells. Furthermore, TIGIT blockage also increased apoptosis and changed the cell cycle distribution of colorectal cancer cells. These results confirmed that blocking the TIGIT signaling pathway in CD3^+^ T cells enhanced the Akt/mTOR pathway activity, thereby improving metabolic activity and its anti-tumor effect.

It has been reported that PD-1 blocks the Akt/mTOR signaling pathway (33, 34) and inhibits the glycolysis of CD8^+^ T cells in gastric cancer (35). These immune checkpoints may affect common signaling pathways in regulating T cell function to achieve synergistic effects. In this study, combined blocking of TIGIT and PD-1 showed further tumor control, decreased reduction of key factors in glucose metabolism, and significantly inhibit Akt/mTOR signaling pathway compared with either single blocking in tumor-bearing mice. Furthermore, combined treatment of TIGIT and PD-1 mAb had achieved good therapeutic effects in other cancers, such as glioblastoma (19), head and neck squamous cell carcinoma (21) and renal cell carcinoma (36). These findings suggest the potential of combined immunotherapy for cancer treatment, which is now receiving increased attention. TIGIT blockade alone or in combination with PD-1 may be a potential strategy for treating colorectal cancer. However, the synergetic mechanism is still unclear. TIGIT could directly bind CD226 in cis, disrupting its homodimerization and binding capacity to CD155 (37). PD-1 induces SHP2-mediated CD226 dephosphorylation, supporting the need for dual PD-1/TIGIT blockade to promote CD226 signaling (38).

In summary, with further research on the immunosuppressive receptors on T cells, an increasing number of results have confirmed that the immunosuppressive receptor TIGIT expressed on the T cell membrane can bind to its ligand on the tumor-specific target cells, inhibit the immune function and promote the escape of tumor cells. Our study illustrates the specific mechanism of TIGIT in immunotherapy of colorectal cancer. Our results suggest that blocking TIGIT and restoring T cell metabolic activity may represent a possible approach to immunotherapy against colorectal cancer.

## Data Availability Statement

The original contributions presented in the study are included in the article/**Supplementary Material**. Further inquiries can be directed to the corresponding author.

## Ethics Statement

The studies involving human participants were reviewed and approved by The Third Affiliated Hospital of Soochow University. The patients/participants provided their written informed consent to participate in this study. The animal study was reviewed and approved by The Third Affiliated Hospital of Soochow University.

## Author Contributions

QS performed the experimentation and wrote the manuscript. LW and MY prepared figures. XJ conducted the statistical analysis. XJ and ZC edited the manuscript. CW conducted the project. All authors contributed to the article and approved the submitted version.

## Funding

This work was supported by Nantong Science and Technology Fund Project (JCZ19040 and JC2020071).

## Conflict of Interest

The authors declare that the research was conducted in the absence of any commercial or financial relationships that could be construed as a potential conflict of interest.

## Publisher’s Note

All claims expressed in this article are solely those of the authors and do not necessarily represent those of their affiliated organizations, or those of the publisher, the editors and the reviewers. Any product that may be evaluated in this article, or claim that may be made by its manufacturer, is not guaranteed or endorsed by the publisher.

## Supplementary Material

Supplementary Figure 1 The effect of TIGIT blockade on the glucose metabolic activity of CD3^+^TIGIT^-^ T cells. **(A)** mRNA expression of Glut1, HK1, HK2, and PFK elevated in TIGIT Ab group compared with the control group in CD3^+^TIGIT^-^ T cells. **(B, C)** Western blotting analysis of the phosphorylation levels of the AKT/mTOR pathway in the control group and TIGIT Ab group in CD3^+^TIGIT^-^ T cells.
