# Supplementary figures and images for: TIGIT Induces (CD3+) T Cell Dysfunction in Colorectal Cancer by Inhibiting Glucose Metabolism
Source: Front Immunol. 2021 Sep 29;12:688961. doi: 10.3389/fimmu.2021.688961 (PMC8511404; doi:10.3389/fimmu.2021.688961)

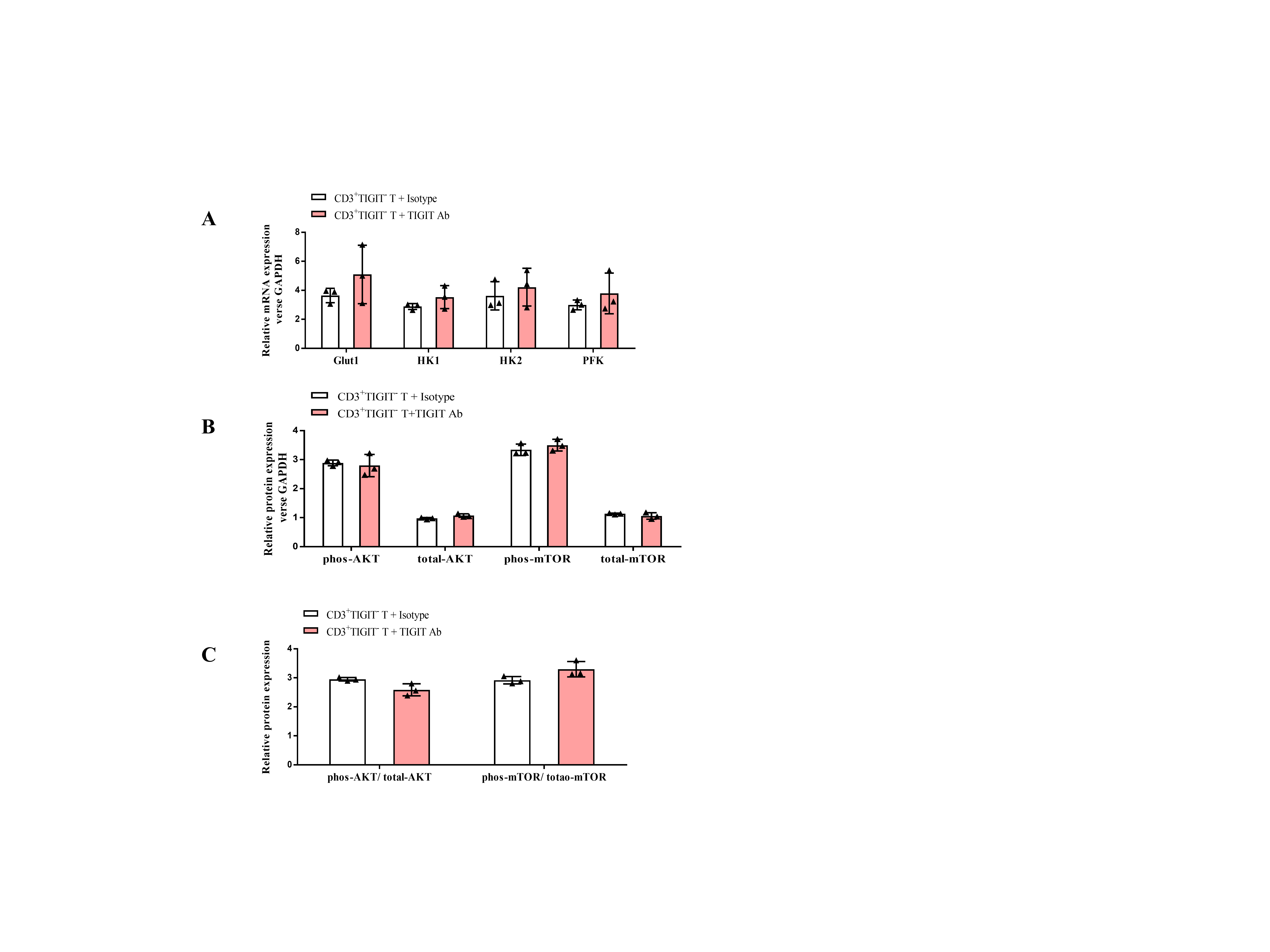

Supplement: Supplementary Figure 1 — The effect of TIGIT blockade on the glucose metabolic activity of CD3+TIGIT- T cells. (A) mRNA expression of Glut1, HK1, HK2, and PFK elevated in TIGIT Ab group compared with the control group in CD3+TIGIT- T cells. (B, C) Western blotting analysis of the phosphorylation levels of the AKT/mTOR pathway in the control group and TIGIT Ab group in CD3+TIGIT- T cells. [file Image_1.tif]
